# Turning a polystyrene microsphere into a multimode light source by laser irradiation

**DOI:** 10.1515/nanoph-2022-0380

**Published:** 2022-10-21

**Authors:** Shimei Liu, Shaolong Tie, Jingdong Chen, Guangcan Li, Jiaxin Yang, Sheng Lan

**Affiliations:** Guangdong Provincial Key Laboratory of Nanophotonic Functional Materials and Devices, School of Information and Optoelectronic Science and Engineering, South China Normal University, Guangzhou 510006, China; School of Chemistry, South China Normal University, Guangzhou 510006, China; College of Physics and Information Engineering, Minnan Normal University, Zhangzhou 363000, China

**Keywords:** laser irradiation, microsphere, polystyrene, two-dimensional material, whispery gallery mode

## Abstract

Polystyrene (PS) is generally considered as a passive optical material that is transparent to light with wavelengths longer than 300 nm. In practice, PS micro- and nanospheres with uniform sizes are usually used to build photonic crystals based on self-assembly mechanism. Here, we demonstrate experimentally that PS microspheres supporting whispery gallery modes can be transformed into multimode light sources by laser irradiation. We show that a PS microsphere placed on a silica substrate can be lighted up when it is consecutively irradiated by using a 488-nm continuous wave laser beam with a pumping power above a threshold. Broadband luminescence emitted from the PS microsphere increases rapidly to a maximum value and decreases gradually with increasing irradiation time, implying the generation and degradation of a certain luminescent material upon laser irradiation. However, the PS microsphere is found to be damaged by high temperature based on morphology examination. By replacing the silica substrate with a thin silver film, the threshold laser power for lighting up a PS microsphere is dramatically reduced. More importantly, we can see enhanced luminescence intensities from the whispery gallery modes supported by the PS microsphere, which becomes an efficient multimode light source. Interestingly, the threshold laser power can be further lowered by inserting a molybdenum disulfide monolayer in between the PS microsphere and the silver film. As a result, the PS microsphere remains nearly unchanged except the formation of the luminescence material. Our findings open a new horizon for the interaction of polymer with laser light by exploiting the optical resonances supported by micro- and nanoparticles and pave the way for constructing photonic devices based on laser-induced luminescent materials in polymers.

## 1 Introduction

Pure polystyrene (PS) is generally considered as a stable optical material in the visible to near infrared spectral range because it does not absorb light with wavelengths longer than 300 nm [1–3]. For this reason, PS nanospheres (NSs) with uniform sizes are usually used to build photonic crystals via the self-assembly mechanism [4, 5]. In addition, PS microspheres (MSs) are commonly employed as optical resonators because they support whispery gallery modes (WGMs) with high quality (Q) factors [6–8]. Since PS is a passive optical material, active devices are usually constructed by using PS films, PS MSs, or PS NSs doped with other luminescent materials [9, 10]. For example, the nonlinear optical responses can be enhanced in Cd/PS composite films [11]. In particular, PS MSs doped with luminescent materials are quite suitable for preparing microlasers with ultralow threshold [12], and exhibit many interesting optical phenomena such as 4-photon excited state absorption [13] and optical bi-stability. Very recently, it was demonstrated that the photoluminescence (PL) or Raman spectra of PS MSs could be enhanced by exploiting plasmon-WGM coupling [14].

It has been known that photodegradation of PS occurs under the irradiation of ultraviolet light (254 nm). In this case, the excitation of phenyl group, which leads to dehydrogenation from carbon atom to phenyl group, creates conjugated double bonds in the hydrocarbon main chain [15]. For many organic materials, their emission intensities at different wavelengths depend strongly on the length of the conjugated double bonds, whose formation mechanism is very complex. Previously, it was reported that stable luminescent substances could be gradually accumulated by repeatedly exciting a PS film with pulsed laser light [16]. As a result, the PL of the PS film increased slowly and reached a stable value with increasing irradiation time. So far, the luminescent substances generated in the photodegradation of PS remain unknown. However, it was suggested that they were formed through the reaction of PS with electrons and holes or by the heat generated by the nonradiative recombination of carriers. In the latter case, the formation process should be related to temperature and oxygen [16]. Since electrons and holes are involved in the formation of the luminescent substances, it was suggested that the multiphoton absorption of the excitation laser light in PS plays a crucial role in the generation of luminescent materials.

Very recently, micro-lasers based on two-dimensional transition metal dichalcogenides (TMDCs) and MSs have attracted great interest because of their excellent properties and broad applications [17–19]. Basically, a PS MS acts as an excellent optical resonator that supports WGMs related to its diameter and refractive index [6, 7, 20]. Since WGM modes are quite sensitive to the surrounding environment, the shift, splitting and broadening of WGMs can be exploited to detect the small change of the surrounding environment [21], which implies potential applications in micro-optical biosensors and temperature sensors [22–25]. More importantly, the WGMs supported by PS MSs can be used to enhance light–matter interaction, making PS MSs a promising candidate for two-dimensional exciton laser [19, 26]. On the other hand, TMDCs possess large exciton resonance intensity and strong quantum confinement effect [27, 28]. The exciton density in a TMDC excited by light can reach a large value of 10^18^ cm^−3^, supporting long-lived population inversion necessary for stimulated emission and optical gain required for lasing [26]. Many recent studies focused on the composite cavities formed by TMDC monolayers and dielectric MSs [29]. Such cavities take the advantage of reduced excitation area due to the lensing effect of the MSs, increasing the coupling efficiency between the excitons in TMDC monolayers and the WGMs supported by the MSs [19]. As a result, the spontaneous emission of excitons is enhanced and modulated by the WGM modes, realizing microlasers [18]. Therefore, PS MSs are promising candidates for developing novel optical devices, such as microlasers, owing to the existence of WGM modes with high quality factors [29].

In this work, we investigate systematically the generation of luminescent materials in PS MSs by laser irradiation. It is found a certain luminescent material can be introduced in a PS MS by irradiating the PS MS with 488-nm continuous wave laser light. A luminescence burst is observed when the pumping power exceeds a critical value (i.e., a threshold). The consecutive irradiation of the PS MS leads to the reduction of the luminescence and the damage of the PS MS. The threshold laser power can be greatly reduced by placing the PS MS on a thin silver (Ag) film due to the enhanced electric field of the WGM mode. By inserting a molybdenum disulfide (MoS_2_) monolayer in between the PS MS and the Ag film, the threshold laser power can be further reduced, leading to the formation of a multimode light source. Our findings facilitate the further development of microlasers and microsensors based on laser-induced lighting of PS MSs.

## 2 Results and discussion

**Figure 1:**
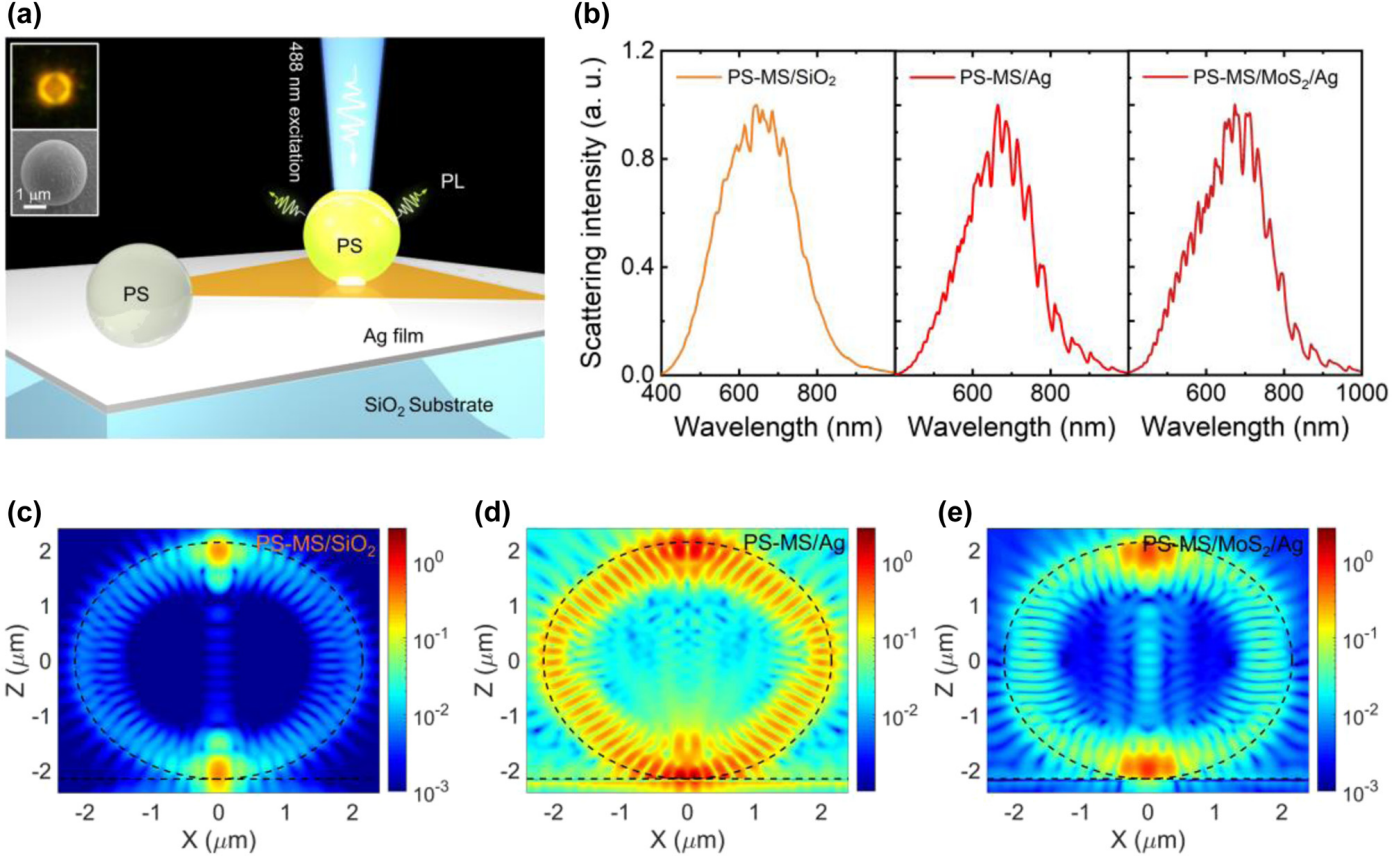
WGMs supported by a SiO_2_ MS placed on different substrates. (a) Schematic showing the characterization of the PL from a PS-MS/MoS_2_/Ag microcavity being excited by an external laser light. The dark-field image and SEM image of a typical PS MS are shown in the insets. (b) Backward scattering spectra measured for a PS-MS/SiO_2_ microcavity, a PS-MS/Ag microcavity, and a PS-MS/MoS_2_/Ag microcavity with *d* ∼ 4.3 μm. (c)–(e) In-plane electric field intensity distribution (|*E*_*xz*_/*E*_0_|) calculated at resonances of 593.8, 590.0, and 595.8 nm for the PS-MS/SiO_2_ microcavity, the PS-MS/Ag microcavity, and the PS-MS/MoS_2_/Ag microcavity with *d* = 4.3 μm, respectively.

In Figure 1a, we show schematically the configurations of three structures studied in this work: (1) a PS MS placed on a silica (SiO_2_) substrate; (2) a PS MS placed on a thin Ag film (or an Ag/SiO_2_ substrate); (3) a PS MS placed on a thin Ag film with an embedded MoS_2_ monolayer. In the following, the three microcavities are denoted as PS-MS/SiO_2_, PS-MS/Ag, and PS-MS/MoS_2_/Ag microcavities, respectively. Here, the diameter of the PS MS is *d* ∼ 4.3 μm and the thickness of the Ag film is 50 nm. In all cases, PS MSs are irradiated by using 488-nm continuous wave laser light with adjustable laser power. The images of a typical PS MS under a dark-field microscope and a scanning electron microscope (SEM) are shown in the insets. In Figure 1b, we present the backward scattering spectra measured for a PS-MS/SiO_2_ microcavity, a PS-MS/Ag microcavity, and a PS-MS/MoS_2_/Ag microcavity. In the scattering spectrum of the PS-MS/SiO_2_ microcavity, only the WGMs at the scattering peak are excited. In comparison, the WGMs located on the short- and long-wavelength side of the scattering peak are also excited in the PS-MS/Ag and PS-MS/MoS_2_/Ag microcavities. In addition, the WGMs appear to be clearer and sharper, which is a result of the enhanced electric field in the PS-MS/Ag and PS-MS/MoS_2_/Ag microcavities, as demonstrated later. For the three microcavities, we examined the electric field distribution in the *xz* plane (i.e., |*E*_*xz*_/*E*_0_|) for a WGM mode close to the scattering peak, as shown in Figure 1c–e. In each case, we used a dipole source oriented along the *x* direction and placed at the contacting point between the PS MS and the substrate to excite the WGM mode. As expected, the electric field of the WGM mode is mainly distributed at the edge of the PS MS. It is noticed, however, that the electric field is enhanced by a factor of ∼8.0 in the PS-MS/Ag microcavity as compared with that in the PS-MS/SiO_2_ microcavity. Although the enhancement factor is reduced dramatically in the PS-MS/MoS_2_/Ag microcavity due mainly to the coupling between the excitons in the MoS_2_ monolayer and the WGM mode, an enhancement factor of ∼2.0 is still achieved. More importantly, a strong localization of the electric field is observed at the contacting point between the PS MS and the Ag film in the PS-MS/Ag microcavity (see Figure 1d). Once a luminescent material is generated in the PS MS, the spontaneous emission will be greatly enhanced at the contact point in the PS-MS/Ag microcavity due to the Purcell effect. In addition, the good conductivity of the Ag film will alleviate the damage of the PS MS by the heat released in the nonradiative recombination process [30].

**Figure 2:**
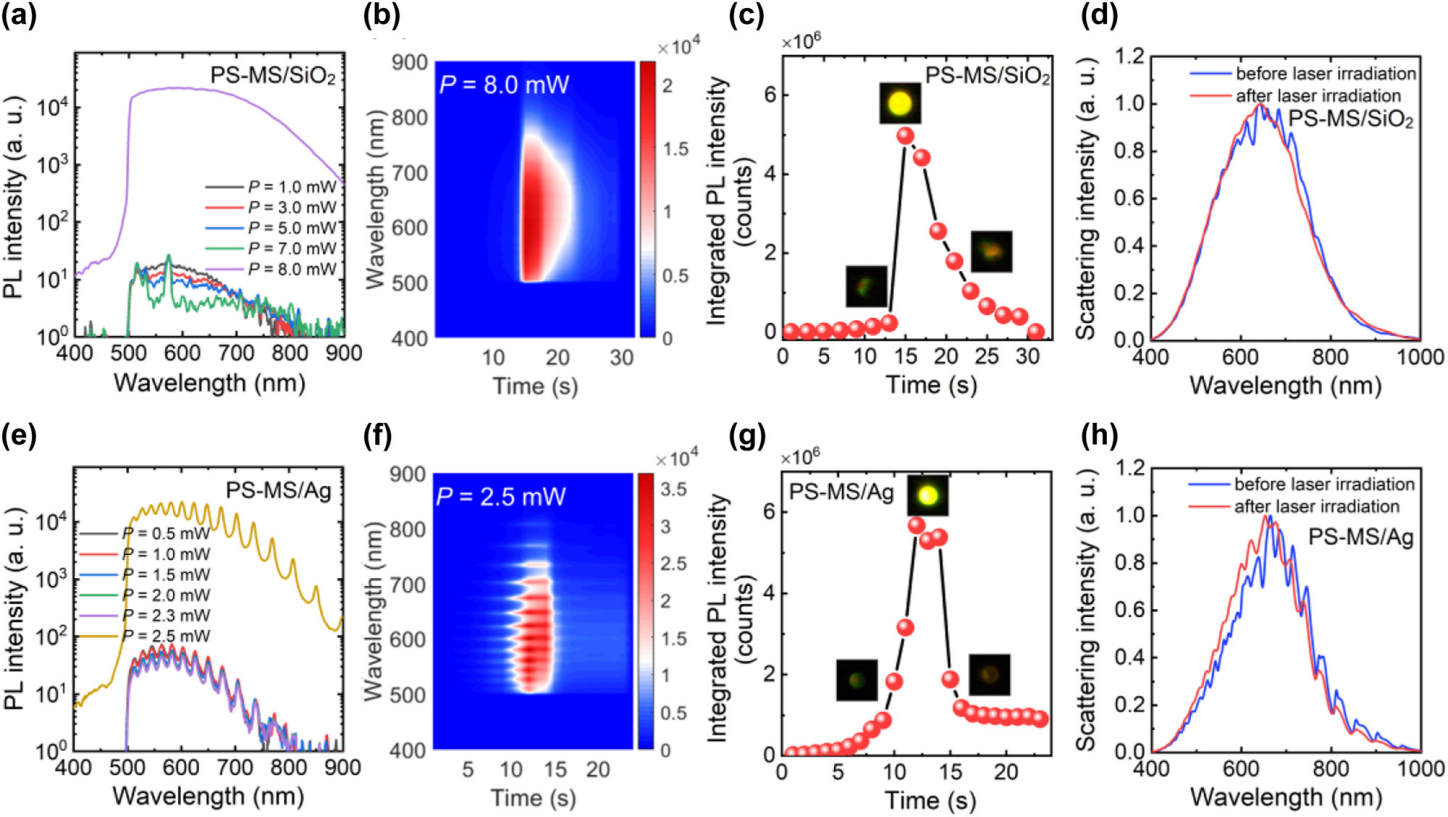
Dramatic increase in PL intensity induced by laser irradiation in a PS-MS/SiO_2_ and a PS-MS/Ag microcavity. (a) PL spectra measured for a PS-MS/SiO_2_ microcavity at different laser powers. (b) Evolution of the PL spectrum with increasing irradiation time measured for the PS-MS/SiO_2_ microcavity excited by using 488-nm laser light at 8 mW. (c) Dependence of the integrated PL intensity of the PS-MS/SiO_2_ on the irradiation time. The images of the PS-MS/SiO_2_ microcavity recorded by using a CCD at different irradiation times are shown in the insets. (d) Scattering spectra measured for the PS-MS/SiO_2_ microcavity before (blue curve) and after (red curve) laser irradiation. (e) PL spectra measured for a PS-MS/Ag microcavity at different laser powers. (f) Evolution of the PL spectrum with increasing irradiation time measured for the PS-MS/Ag microcavity excited by using 488-nm laser light at 2.5 mW. (g) Dependence of the integrated PL intensity of the PS-MS/Ag microcavity on the irradiation time. The images of the PS-MS/Ag microcavity recorded by using a CCD at different irradiation times are shown in the insets. (h) Scattering spectra measured for the PS-MS/Ag microcavity before (blue curve) and after (red curve) laser irradiation.

It is well known that PS is quite stable against light with wavelengths longer than 300 nm and one-photon absorption by PS in the visible light spectrum is negligible. For a PS MS supporting WGM modes, however, the above conclusion is no longer valid because the enhanced light–matter interaction mediated by the WGM modes. In this work, we irradiated the PS-MS/SiO_2_ microcavity with a 488-nm laser beam and observed weak luminescence coming from the microcavity, as shown in Figure 2a. At low laser powers (*P* = 1.0 and 3.0 mW), we observed broadband luminescence without any feature of the WGM modes. When the laser power is raised to *P* = 5.0 mW, small peaks emerge in the spectrum. This phenomenon becomes more apparent for *P* = 7.0 mW. As the laser power is increased to *P* = 8.0 mW, a dramatic increase in the luminescence intensity by more than three orders of magnitude is observed, as shown in Figure 2a. It should be emphasized, however, that the dramatic increase in the luminescence does not appear immediately after the microcavity is irradiated. Instead, it occurs suddenly after the microcavity is irradiated consecutively by the laser light for some time, as shown in Figure 2b. It is remarkable that the luminescence intensity reaches a maximum value rapidly and decreases gradually if the laser irradiation continues. If the rapid increase of the luminescence intensity is induced by the quick generation of the luminescent material in an avalanche process, then the gradual reduction of the luminescence intensity is probably caused by the thermal- or photo-decomposition of the luminescent material. It is noticed that the luminescence spectrum at the maximum intensity appears to be broad and flat and the small peaks appearing in the spectra at low laser powers disappear completely (see Figure 2a). If we examine the evolution of the integrated PL intensity with increasing irradiation time, which is shown in Figure 2c, it is found that the integrated PL intensity reaches a maximum value rapidly and decreases gradually with increasing irradiation time. This behavior is commonly observed when the laser power exceeds a critical value (or a threshold). The rapid increase of the luminescence intensity is induced by the quick generation of the luminescent material in an avalanche process while the gradual reduction of the luminescence intensity is probably caused by the thermal- or photo-decomposition of the luminescent material. In the insets of Figure 2c, we provide the images of the microcavity recorded by using a charge coupled device (CCD) at different times, which show clearly the lighting and quenching of the microcavity upon the laser irradiation.

**Figure 3:**
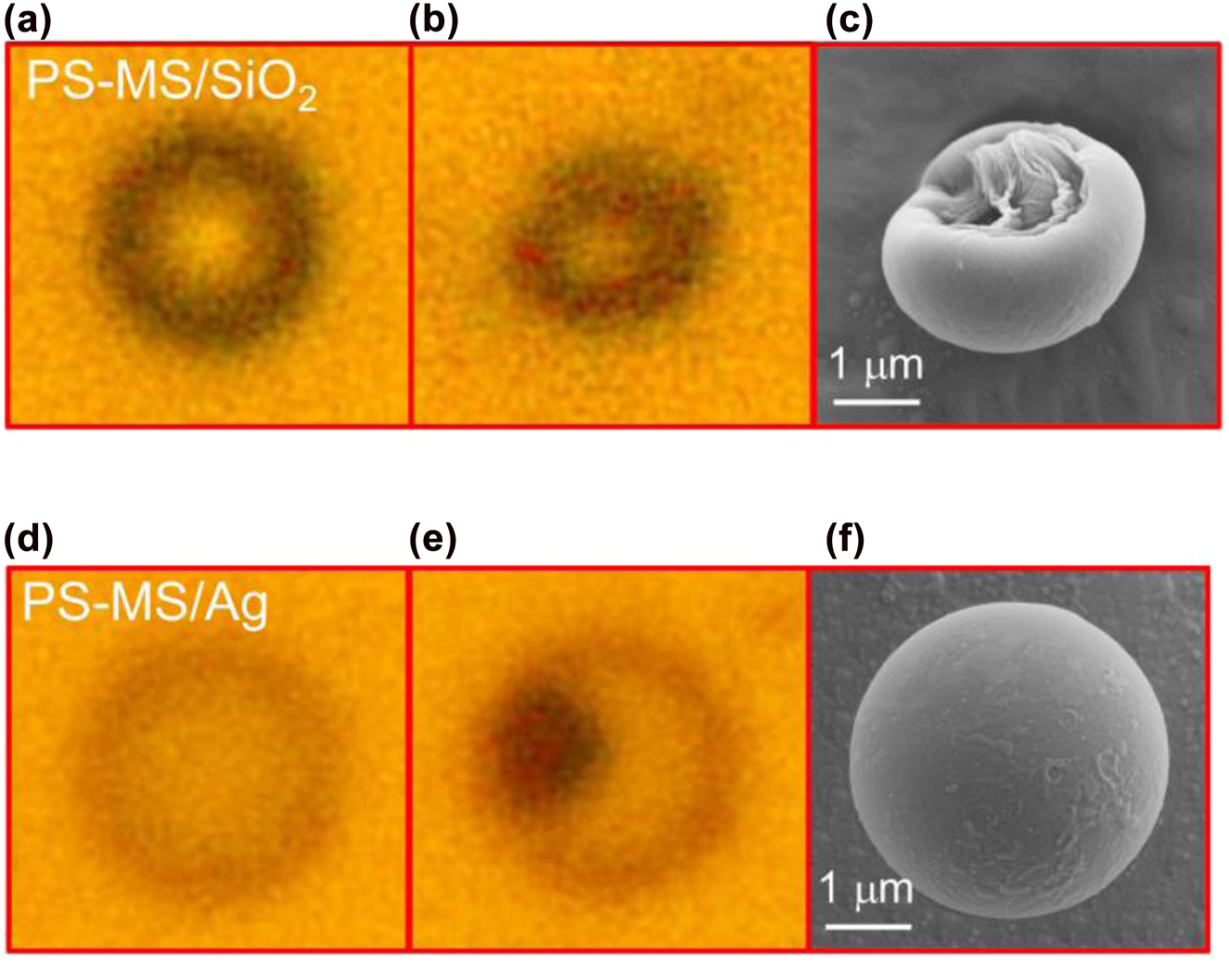
Bright-field images of the PS-MS/SiO_2_ microcavity before (a) and after (b) laser irradiation. (c) SEM image of the PS-MS/SiO_2_ microcavity after laser irradiation. The bright-field images of the PS-MS/Ag microcavity before and after laser irradiation are shown in (d) and (e). (f) SEM image of the PS-MS/Ag microcavity after laser irradiation.

In order to determine whether the PS MS is damaged after the laser excitation, we compared the backward scattering spectra of the PS MS before and after the laser irradiation, as shown in Figure 2d. It can be seen that the small peaks in the scattering spectrum, which represent the WGM modes supported by the PS MS, disappear completely after the laser irradiation. This behavior implies that the PS MS may be damaged in the lighting process, probably by the heat generated in the nonradiative recombination process. This suspect is confirmed by the optical and morphology examinations based on bright-field microscopy and SEM, as shown in Figure 3a–c. Before the laser irradiation, the PS MS appears as a dark circle under the bright-field microscope (see Figure 3a). After the laser irradiation, the circular shape is distorted obviously, implying the damage of the PS MS (see Figure 3b). The damage of the PS MS is verified by the SEM image shown in Figure 3c, where a collapse crater is observed in the PS MS. The formation of the collapse crater explains why the WGM modes disappear in both the luminescence and the scattering spectra.

The large threshold (*P* = 8 mW) for lighting up the PS-MS/SiO_2_ microcavity results in a high temperature that leads to the formation of collapse crater in the PS MS. In order to overcome this shortage, one should improve the interaction of a PS MS with light via the localization of the electric field. In this work, we replaced the SiO_2_ substrate with an Ag/SiO_2_ substrate, creating PS-MS/Ag microcavities, as schematically shown in Figure 1a. As discussed above, the electric field of the WGM mode can be enhanced by a factor of ∼8.0 in a PS-MS/Ag microcavity (see Figure 1d). We excited a PS-MS/Ag microcavity with the laser light and examined the luminescence of the microcavity with increasing laser power. As shown in Figure 2e, the luminescence intensity remains nearly unchanged when the laser power is lower than *P* = 2.3 mW. Differently, the enhanced luminescence intensities at the WGM modes supported by the microcavity are clearly observed in the luminescence spectra, even at a low laser power of *P* = 0.5 mW. This feature implies the enhanced spontaneous emission rates achieved at the WGM modes due to the Purcell effect. As expected, a significant increase in the luminescence intensity by more than three orders of magnitude is observed when the laser power is raised to *P* = 2.5 mW, as shown in Figure 2e. In this case, one can see narrow and sharp peaks in the PL spectrum of the PS-MS/Ag microcavity, which are WGMs amplified by the gain of the luminescent material. The WGM modes of the microcavity with quality factors of ∼47 are clearly identified in the luminescence spectrum. It is noticed that the luminescence intensity increases gradually and decreases rapidly as compared with the PS-MS/SiO_2_ microcavity. We think that the good thermal conductivity of Ag and the enhanced electric field are responsible for these differences. Basically, the thermal accumulation effect is more pronounced in the PS-MS/SiO_2_ microcavity due to the low thermal conductivity of SiO_2_. As a result, the luminescent material is generated in an avalanche process in the PS-MS/SiO_2_ microcavity, leading to a rapid increase of the luminescent intensity. In comparison, the temperature rise in the PS-MS/Ag microcavity is slower because of the good thermal conductivity of Ag. However, it is expected that the decomposition of the luminescent material is faster in the PS-MS/Ag microcavity due to the enhanced electric field. Unfortunately, a gradual reduction of the luminescence intensity is still observed if the microcavity is consecutively irradiated by the laser light with *P* = 2.5 mW, as show in Figure 2f. In the Figure 2g, we show the integrated PL intensity of the microcavity as a function of the irradiation time. Similarly, we observe a rapid increase of the PL intensity followed by a gradual decrease with increasing irradiation time. We also compared the backward scattering spectra of the PS-MS/Ag microcavity before and after the laser irradiation, as shown in Figure 2h. It is noticed that the WGM modes appearing in the scattering spectrum are smeared after the laser excitation. We also examined the optical images of the PS MS before and after the laser irradiation under the bright-field microscope, as shown in Figure 3d and e. A dark region corresponding to a large extinction in the visible light spectrum is observed after the laser excitation. We suspect that the dark region originates from the decomposition of the luminescent material. Surprisingly, we did not observe the damage of the PS MS based on the SEM observation, as shown in Figure 3f. This is because that the threshold for lighting up the PS MS is greatly reduced due to the introduction of the thin Ag film, which not only enhances the spontaneous rates at the WGM modes but also serves as a good heat sink.

**Figure 4:**
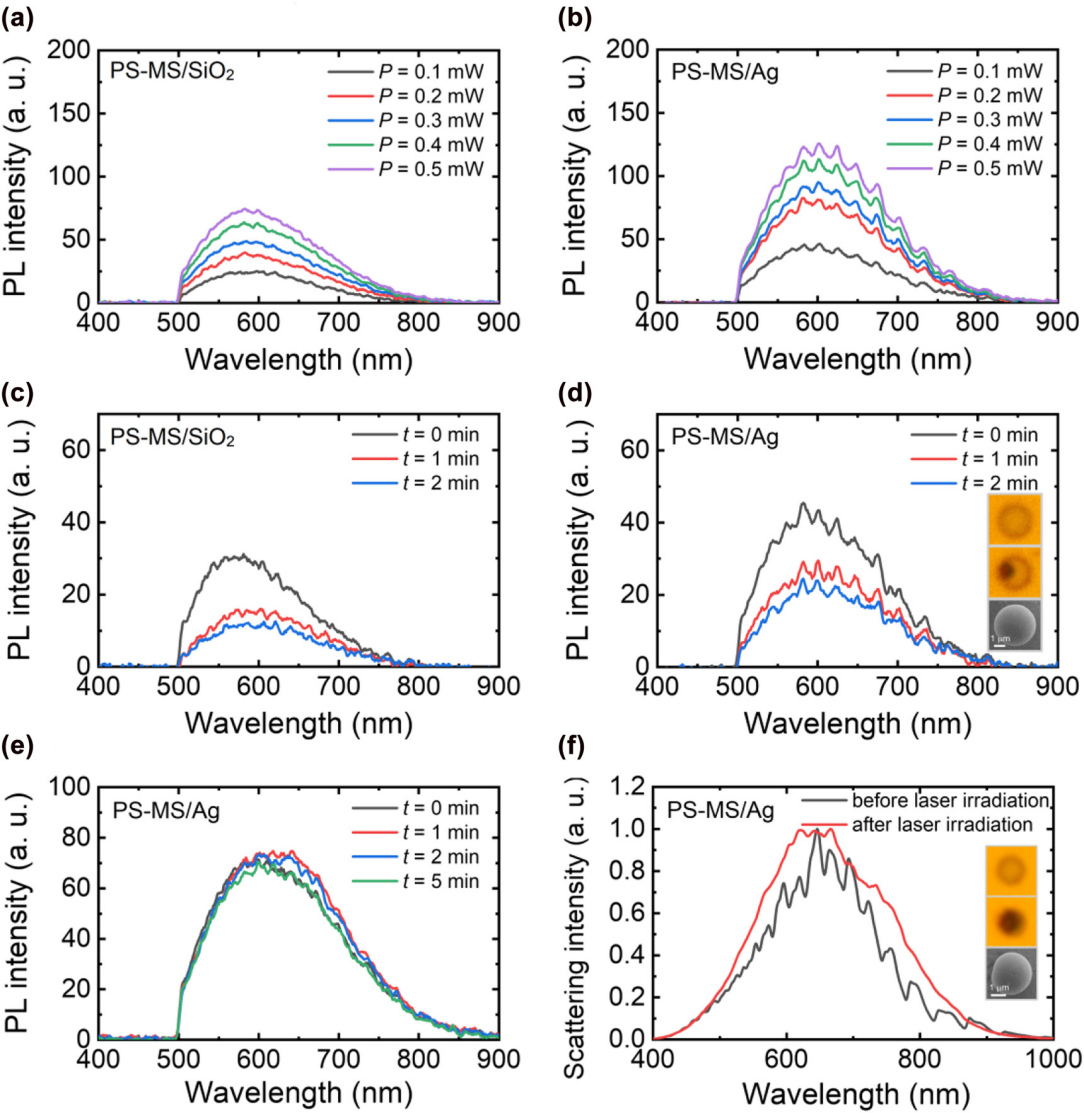
PL spectra measured at low laser powers for the PS-MS/SiO_2_ microcavity (a) and the PS-MS/Ag microcavity (b) shown in Figure 2a and e. The evolution of the PL spectrum with increasing irradiation time measured for the PS-MS/SiO_2_ microcavity and the PS-MS/Ag microcavity at a laser power of *P* = 0.05 mW are shown in (c) and (d). (e) Evolution of the PL spectrum with increasing irradiation time measured for another PS-MS/Ag microcavity at a laser power of *P* = 0.05 mW. (f) Scattering spectra measured for the PS-MS/Ag microcavity shown in (e) before (black curve) and after (red curve) laser irradiation. The bright-field images of the PS-MS/Ag microcavity before and after laser irradiation and the SEM image of the PS-MS/Ag microcavity after laser irradiation are shown in the insets.

In order to find out the changes in the luminescence properties of PS-MS/SiO_2_ and PS-MS/Ag microcavities induced by laser irradiation, we re-excited the two samples in the low excitation power regime. As shown in Figure 4a and b, the PL intensity increases gradually with increasing laser power, implying the generation of luminescent material after the laser excitation. This behavior is different from that observed before the luminescence burst, which is presented in Figure 2a and e. In that case, no obvious increase of PL intensity is observed. This luminescent material may be associated with the breaking of chemical single bonds and the creation of conjugated double bonds which are contained in the phenyl and the main chain. In Figure 4c and d, we show the evolution of the PL spectrum with irradiation time recorded at a laser power of *P* = 0.05 mW. In both cases, we observed the reduction of the PL intensity under laser excitation. It implies that the luminescent material is not stable against laser irradiation and it may be decomposed by the heat accumulation. In Figure 4d, one can see a dark spot in the PS MS, which is caused by the decomposition of the luminescent material. The decomposition of the luminescent material in other place, which results in the reduction in the PL intensity, may occur under laser irradiation. In Figure 4e, we show the evolution of the PL spectrum with irradiation time measured for another PS-MS/Ag at a laser power of *P* = 0.05 mW. In this case, the PL intensity remains almost unchanged with increasing irradiation time. In addition, the WGM modes appearing in the PL spectra become unclear as compared with those shown in Figure 4d. If we compare the scattering spectra of the PS-MS/Ag before and after the laser irradiation, it is noticed that the WGM modes revealed in the scattering spectrum of PS-MS/Ag disappear completely after the laser excitation (see Figure 4f). As we examined the bright-field image of the PS-MS/Ag, it is found that the entire PS MS becomes black after the first luminescence burst, as shown in the inset of Figure 4f. In this case, the decomposition of the luminescent material induced by laser irradiation is not significant. As a result, the PL intensity remains almost unchanged. Physically, the disappearance of the WGMs may be caused either by the surface roughening of the PS MS or by the absorption of the generated luminescent material. The first factor is excluded by inspecting the surface of the PS MS based on SEM, as shown in the inset of Figure 4f. Thus, we believe that the decomposition of the luminescent material is responsible for the vanishing of the WGM modes after the laser excitation.

**Figure 5:**
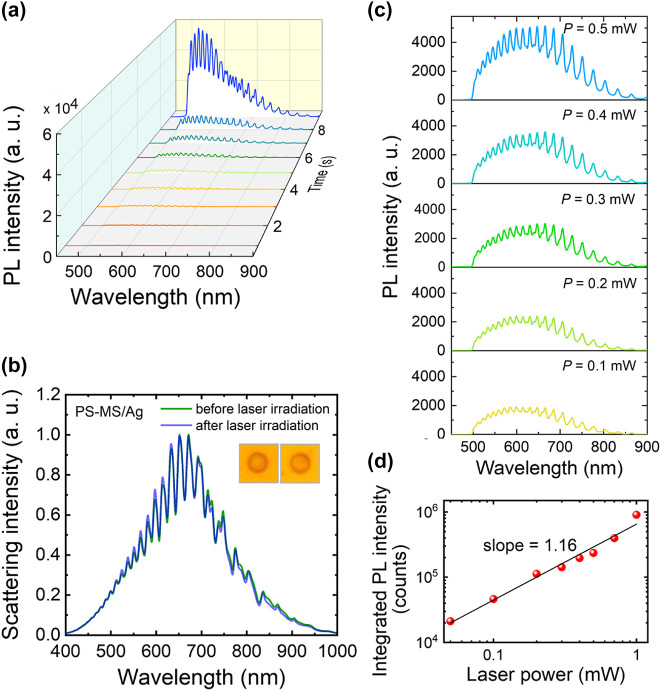
Emission from the WGMs supported by a PS-MS/Ag microcavity. (a) Evolution of the PL spectrum of a PS-MS/Ag microcavity with increasing irradiation time measured at a laser power of *P* = 2.5 mW. (b) Scattering spectra measured for the PS-MS/Ag microcavity before (green curve) and after (purple curve) laser irradiation. The bright-field images of the PS-MS/Ag microcavity before and after laser irradiation are shown in the insets. (c) PL spectra measured for the PS-MS/Ag microcavity at different laser powers below the threshold. (d) Dependence of the integrated PL intensity of the PS-MS/Ag microcavity on the laser power.

As discussed above, the luminescent material induced by laser irradiation may be decomposed by the heat generated in the rapid growth of the PL intensity, leading eventually to the reduction of the PL intensity and the disappearance of the WGM modes. In order to prevent the decomposition of the luminescent material, we can intentionally switch off the laser light when the PL intensity reaches the maximum value. An example is shown in Figure 5a where the evolution of the PL spectrum with increasing irradiation time recorded for a PS-MS/Ag microcavity is presented. In Figure 5b, we show the scattering spectra measured for the PS-MS/Ag microcavity before and after the laser irradiation. Surprisingly, the WGM modes survive after the laser irradiation and the scattering spectrum remains nearly unchanged. Accordingly, no change is observed in the bright-field image of the PS-MS/Ag, as shown in the insets. It implies that the luminescent material induced by the laser irradiation is nearly transparent in the visible light spectrum. In addition, the decomposition of the luminescent material is avoided in this case. We re-excited the PS-MS/Ag in the low power regime, as shown in Figure 5c. It is found that the PL intensity is very strong even at low laser powers and it increases with increasing laser power. In Figure 5d, we show the dependence of the integrated PL intensity on the excitation laser power, which is plotted in a logarithmic coordinate. A slope of ∼1.16 was extracted from the fitting of the experimental data. Basically, the carrier recombination mechanism can be revealed from the relationship between the integrated PL intensity (*I*) and excitation power density (*P*), which can be expressed as *I* ∝ *P* ^*k*^. Here, *k* ≥ 2 is a recombination dominated by free carriers, 1 < *k* < 2 is a recombination of excitons, and *k* < 1 is a free-to-bound recombination [31, 32]. Therefore, the slope extracted from the experimental data suggests that the luminescence of a PS MS originates from the recombination of excitons. Moreover, the enhanced emissions from the WGM modes supported by the PS-MS/Ag microcavity are clearly resolved in the PL spectra. It means that the PS-MS/Ag microcavity has become an efficient multimode light source.

**Figure 6:**
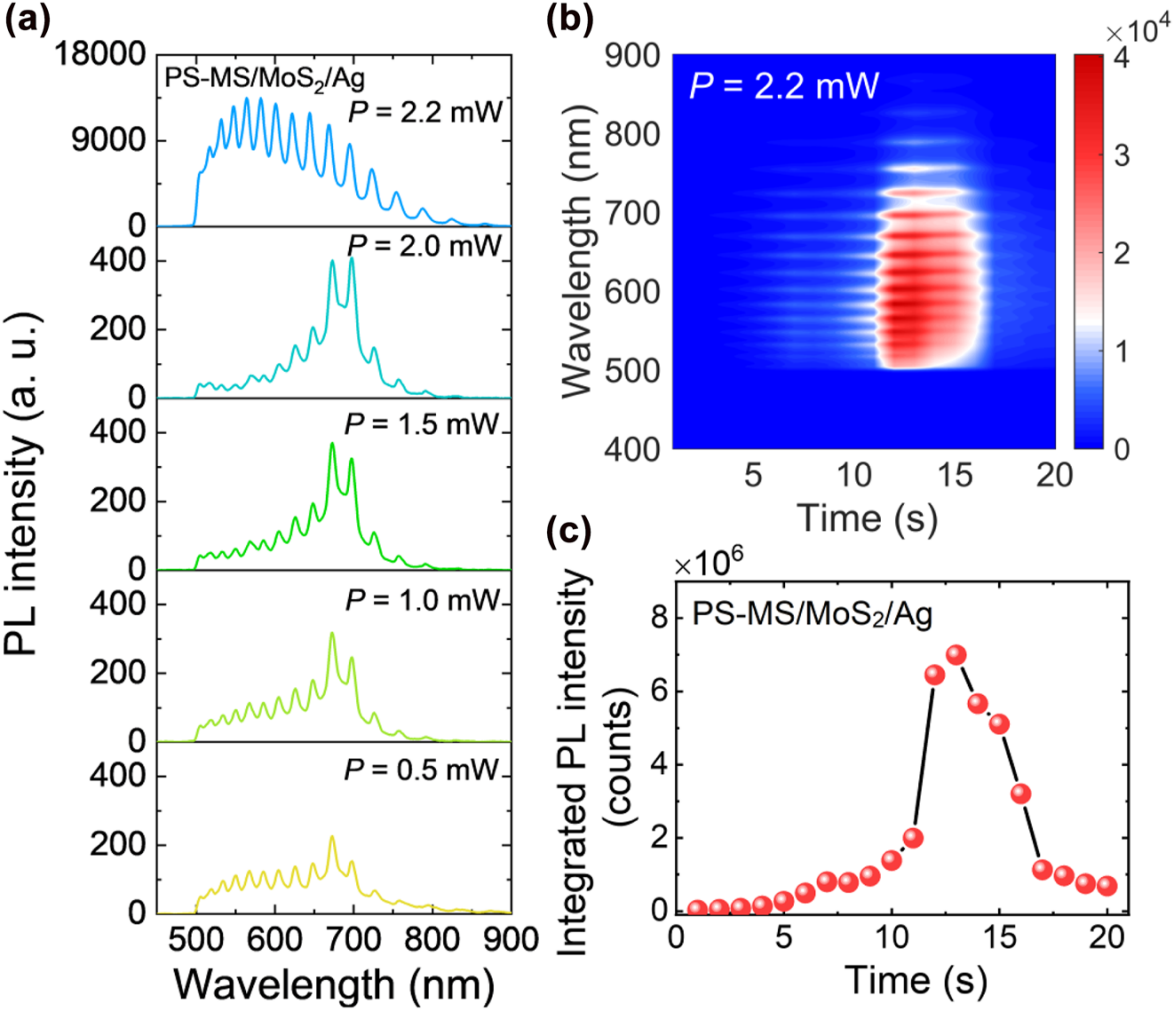
Lighting up a PS-MS/MoS_2_/Ag microcavity by laser irradiation. (a) PL spectra measured for a PS-MS/MoS_2_/Ag microcavity at different laser powers. (b) Evolution of the PL spectrum of the PS-MS/MoS_2_/Ag microcavity with irradiation time measured at a laser power of *P* = 2.2 mW. (c) Dependence of the integrated PL intensity of PS-MS/MoS_2_/Ag microcavity on the irradiation time.

Apparently, the threshold for lighting up a PS MS is crucial for achieving the efficient emissions from the WGM modes of the PS MS. By introducing a two-dimensional material (MoS_2_ monolayer) in between the PS MS and the Ag film, which forms a PS-MS/MoS_2_/Ag microcavity, we can further reduce the threshold. In Figure 6a, we show the PL spectra of the PS-MS/MoS_2_/Ag microcavity measured at different laser powers. At low laser powers (*P* < 2.0 mW), one can identify clearly the WGM modes supported by the PS-MS/MoS_2_/Ag. In addition, the enhanced emission from the exciton resonance of the MoS_2_, which is located at ∼670 nm, is also observed. The coupling between the WGM modes and the exciton resonance is responsible for the enhancement. However, the enhanced emission at the exciton resonance disappears when the laser power is raised to *P* = 2.2 mW. In this case, only WGM modes are revealed in the PL spectrum. In Figure 6b, we show the evolution of the PL spectrum with irradiation time when the PS-MS/MoS_2_/Ag microcavity is excited by using a laser power of *P* = 2.2 mW. It is observed that the PL intensity of the PS-MS/MoS_2_/Ag microcavity increases suddenly at *t* = 12 s, reaching the maximum value immediately and diminishing rapidly after several seconds (see also the Figure 6c). This phenomenon is quite similar to that observed in PS-MS/SiO_2_ and PS-MS/Ag microcavities. In this way, the threshold for lighting up the PS-MS is further reduced by exploiting the interaction between the WGM modes and the excitons in MoS_2_ monolayer.

**Figure 7:**
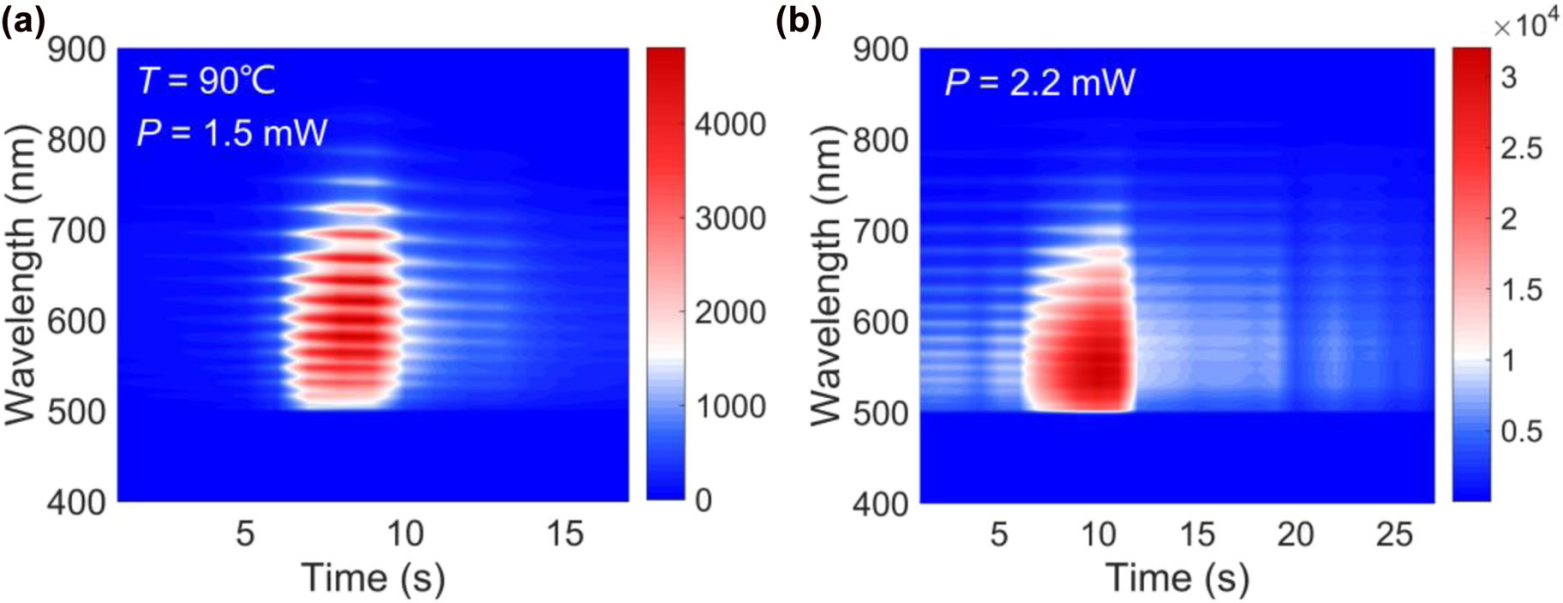
Factors affecting the formation of the laser-induced luminescent material. (a) Evolution of the PL spectrum of a PS-MS/MoS_2_/Ag microcavity with increasing irradiation time observed at *T* = 90 °C and *P* = 1.5 mW in atmosphere. (b) Evolution of the PL spectrum of a PS-MS/MoS_2_/Ag microcavity with increasing irradiation time observed at *P* = 2.2 mW in vacuum.

So far, we have showed that luminescent materials can be induced in a PS MS by irradiating it with laser light. However, there exists a threshold laser power above which the PS MS will become luminescent. Although the physical mechanism for the threshold laser power remains unclear, it has been demonstrated that the threshold can be reduced by placing the PS MS on a thin Ag film and introducing a MoS_2_ monolayer into a PS-MS/Ag microcavity. However, it was found that the PL of the PS MS will increase linearly with the laser power once the luminescent materials are generated by laser irradiation, as shown in Figure 5d. In this case, the PL of the PS MS is similar to that of inorganic materials, such as ceramics [33]. In previous studies, it was suggested that the generation of the luminescent material might be related to oxygen and heat [16]. In order to clarify the factors that affect the formation of the luminescent material in PS induced by laser irradiation, we intentionally increased the environment temperature and examined the threshold power for lighting up a PS-MS/MoS_2_/Ag microcavity. It was found that the threshold power was reduced to *P* = 1.5 mW, as shown in Figure 7a. This result indicates that the generation of the luminescence material is closely related to the heat accumulated in the PS MS. The large absorption of the MoS_2_ monolayer at the exciton resonance plays an important role in the heat generation in PS-MS/MoS_2_/Ag, leading to the reduction of the threshold. We also characterized the threshold power for lighting up a PS-MS/MoS_2_/Ag microcavity placed in a vacuum chamber. In this case, the threshold laser power is found to be *P* = 2.2 mW, which is the same as that observed in air (see the Figure 7b). Therefore, we think that the generation of the luminescent material is not relevant to oxygen.

## 3 Conclusions

In summary, we have constructed PS-MS/Ag and PS-MS/MoS_2_/Ag hybrid microcavities to enhance the interaction of PS with laser light by exploiting the WGM modes supported by such microcavities and the exciton resonance in MoS_2_ monolayer. Different from previous studies, we investigated the luminescent material generated in pure PS MSs without any doping via laser irradiation by exploiting the WGMs supported by the PS MSs. The electric field (|*E*_*xz*_/*E*_0_|) in a PS MS can be enhanced by a factor of ∼8.0 by placing it on a thin Ag film, leading to a dramatic reduction in the threshold for lighting up the PS MS. Moreover, it is revealed that the interaction between the WGM modes and the exciton resonance in MoS_2_ monolayer can further reduce the threshold laser power. The highly efficient PL from PS-MS/Ag and PS-MS/MoS_2_/Ag microcavities with distinct emissions from the WGM modes make them promising multimode light sources. It is found that the generation of the luminescent material is strongly related to the heat accumulation in the PS MS but not relevant to oxygen. Our findings open new horizons for introducing luminescent material in polymers by exploiting the optical resonances supported by polymer MSs and the exciton resonances of two-dimensional materials and pave the way to realize highly efficient multimode light sources for various practical applications.

## 4 Methods

### 4.1 Sample preparation

The MoS_2_ monolayers used in this work were firstly synthesized on a Si substrate via chemical vapor deposition method and then transferred to an Ag/SiO_2_ substrate with a 50-nm-thick Ag film. The aqueous solution of PS microspheres (∼4.3 μm) was dropped and dried on MoS_2_ monolayers attached on the Ag/SiO_2_ substrate, obtaining PS-MS/Ag and PS-MS/MoS_2_/Ag microcavities.

### 4.2 Optical characterization

The scattering spectra of different microcavities were measured by using a dark-field microscope (Axio Observer A1. Zeiss) equipped with a spectrometer (SR-5001-B1. Andor) and a color charge coupled device (CCD) (DS-Ri2. Nikon). For the measurements of the PL spectra, a 488-nm laser light beam was introduced into the microscope and focused on PS-MS/Ag or PS-MS/MoS_2_/Ag microcavities by using a 50× objective.

### 4.3 Numerical simulations

In this work, the numerical simulations were performed by using the finite-difference time-domain (FDTD) technique. The simulation area is three-dimensional. The dielectric constants of Ag and polystyrene were taken from Palik [34] and Sultanova [35], respectively. The dielectric constant of MoS_2_ monolayer was obtained from previous literature [36]. The A exciton energy in MoS_2_ monolayer is 1.83 eV. The refractive index of the surrounding media was chosen to be 1.0. In the calculation, the thickness of the Ag film and MoS_2_ monolayer were chosen to be 50.0 and 1.0 nm. A dipole source oriented along the *x* direction and placed at contacting point between the PS MS and the Ag film in the PS-MS/Ag microcavity is used to excite the WGM mode.
